# Supplementary figures and images for: Adaptive Evolution and Functional Differentiation of Testis-Expressed Genes in Theria
Source: Animals (Basel). 2024 Aug 9;14(16):2316. doi: 10.3390/ani14162316 (PMC11350913; doi:10.3390/ani14162316)

Figure S1

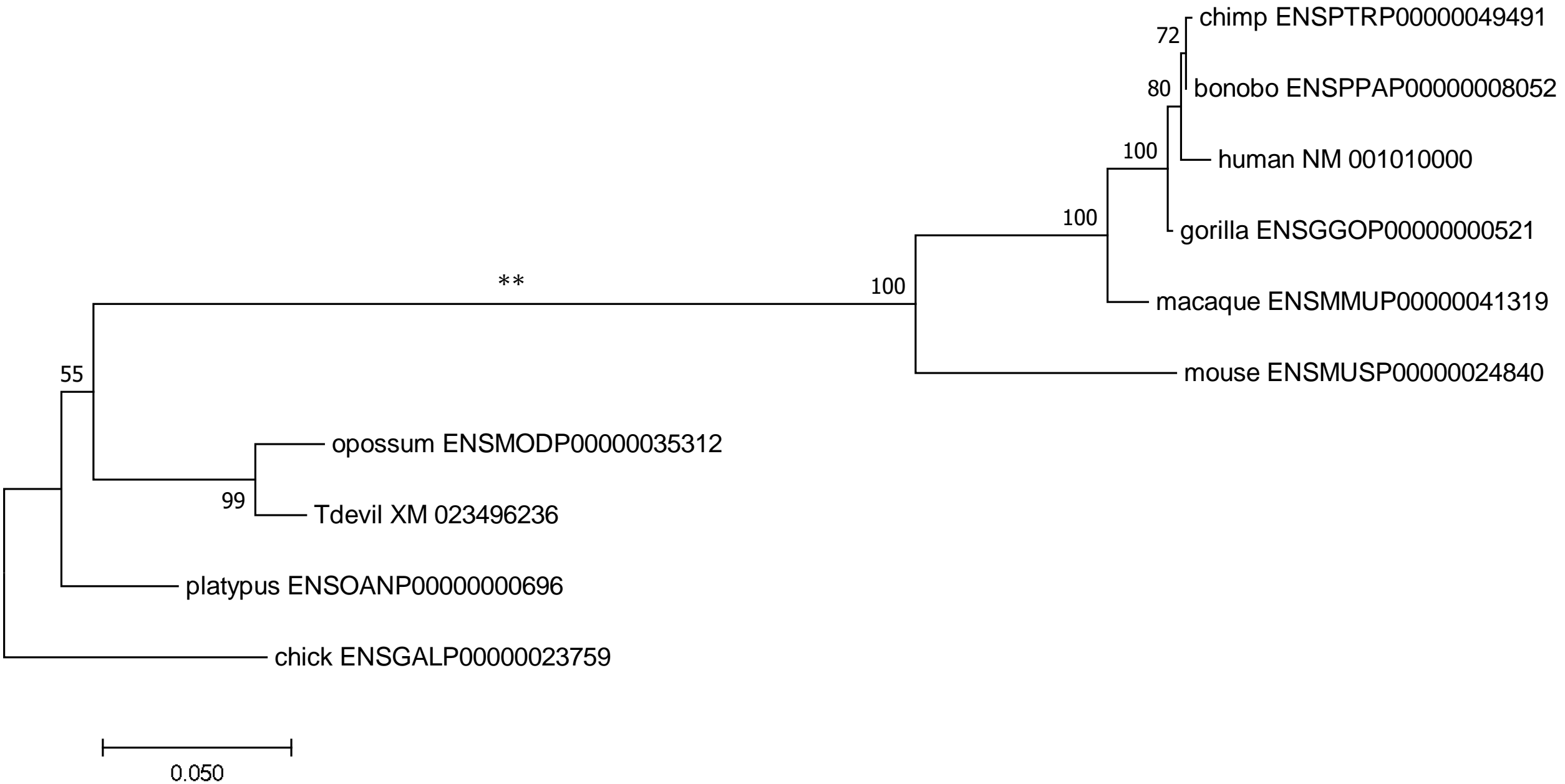

Supplement: Supplementary file 1 [file animals-14-02316-s001.zip › Figure S1.pdf]
